# CanMethdb: a database for genome-wide DNA methylation annotation in cancers

**DOI:** 10.1093/bioinformatics/btac783

**Published:** 2022-12-07

**Authors:** Jianmei Zhao, Fengcui Qian, Xuecang Li, Zhengmin Yu, Jiang Zhu, Rui Yu, Yue Zhao, Ke Ding, Yanyu Li, Yongsan Yang, Qi Pan, Jiaxin Chen, Chao Song, Qiuyu Wang, Jian Zhang, Guohua Wang, Chunquan Li

**Affiliations:** The First Affiliated Hospital, Department of Cardiology, Hengyang Medical School, University of South China, Hengyang 421001, China; College of Life Sciences, Northeast Forestry University, Harbin 150038, China; School of Medical Informatics, Daqing Campus, Harbin Medical University, Daqing 163711, China; The First Affiliated Hospital, Department of Cardiology, Hengyang Medical School, University of South China, Hengyang 421001, China; School of Computer, University of South China, Hengyang 421001, China; The First Affiliated Hospital, Cardiovascular Lab of Big Data and Imaging Artificial Intelligence, Hengyang Medical School, University of South China, Hengyang 421001, China; Department of Cell Biology and Genetics, School of Basic Medical Sciences, Hengyang Medical School, University of South China, Hengyang 421001, China; School of Medical Informatics, Daqing Campus, Harbin Medical University, Daqing 163711, China; The First Affiliated Hospital, Department of Cardiology, Hengyang Medical School, University of South China, Hengyang 421001, China; School of Computer, University of South China, Hengyang 421001, China; The First Affiliated Hospital, Cardiovascular Lab of Big Data and Imaging Artificial Intelligence, Hengyang Medical School, University of South China, Hengyang 421001, China; School of Medical Informatics, Daqing Campus, Harbin Medical University, Daqing 163711, China; College of Information and Computer Engineering, Northeast Forestry University, Harbin 150038, China; College of Bioinformatics Science and Technology, Harbin Medical University, Harbin 150088, China; College of Bioinformatics Science and Technology, Harbin Medical University, Harbin 150088, China; College of Information and Computer Engineering, Northeast Forestry University, Harbin 150038, China; School of Medical Informatics, Daqing Campus, Harbin Medical University, Daqing 163711, China; School of Medical Informatics, Daqing Campus, Harbin Medical University, Daqing 163711, China; Department of Physiology and Pathophysiology, School of Basic Medical Sciences, Department of Rheumatology, Zhon gshan Hospital, Fudan University, Shanghai 200433, China; Shenzhen Bay Laboratory, Pingshan Translational Medicine Center, Shenzhen 518118, China; The First Affiliated Hospital, Department of Cardiology, Hengyang Medical School, University of South China, Hengyang 421001, China; School of Computer, University of South China, Hengyang 421001, China; The First Affiliated Hospital, Cardiovascular Lab of Big Data and Imaging Artificial Intelligence, Hengyang Medical School, University of South China, Hengyang 421001, China; The First Affiliated Hospital, Department of Cardiology, Hengyang Medical School, University of South China, Hengyang 421001, China; School of Computer, University of South China, Hengyang 421001, China; The First Affiliated Hospital, Cardiovascular Lab of Big Data and Imaging Artificial Intelligence, Hengyang Medical School, University of South China, Hengyang 421001, China; School of Medical Informatics, Daqing Campus, Harbin Medical University, Daqing 163711, China; College of Life Sciences, Northeast Forestry University, Harbin 150038, China; College of Information and Computer Engineering, Northeast Forestry University, Harbin 150038, China; The First Affiliated Hospital, Department of Cardiology, Hengyang Medical School, University of South China, Hengyang 421001, China; School of Medical Informatics, Daqing Campus, Harbin Medical University, Daqing 163711, China; School of Computer, University of South China, Hengyang 421001, China; The First Affiliated Hospital, Cardiovascular Lab of Big Data and Imaging Artificial Intelligence, Hengyang Medical School, University of South China, Hengyang 421001, China; Department of Cell Biology and Genetics, School of Basic Medical Sciences, Hengyang Medical School, University of South China, Hengyang 421001, China

## Abstract

**Motivation:**

DNA methylation within gene body and promoters in cancer cells is well documented. An increasing number of studies showed that cytosine–phosphate–guanine (CpG) sites falling within other regulatory elements could also regulate target gene activation, mainly by affecting transcription factors (TFs) binding in human cancers. This led to the urgent need for comprehensively and effectively collecting distinct *cis*-regulatory elements and TF-binding sites (TFBS) to annotate DNA methylation regulation.

**Results:**

We developed a database (CanMethdb, http://meth.liclab.net/CanMethdb/) that focused on the upstream and downstream annotations for CpG–genes in cancers. This included upstream *cis-*regulatory elements, especially those involving distal regions to genes, and TFBS annotations for the CpGs and downstream functional annotations for the target genes, computed through integrating abundant DNA methylation and gene expression profiles in diverse cancers. Users could inquire CpG–target gene pairs for a cancer type through inputting a genomic region, a CpG, a gene name, or select hypo/hypermethylated CpG sets. The current version of CanMethdb documented a total of 38 986 060 CpG–target gene pairs (with 6 769 130 unique pairs), involving 385 217 CpGs and 18 044 target genes, abundant *cis*-regulatory elements and TFs for 33 TCGA cancer types. CanMethdb might help biologists perform in-depth studies of target gene regulations based on DNA methylations in cancer.

**Availability and implementation:**

The main program is available at https://github.com/chunquanlipathway/CanMethdb.

**Supplementary information:**

Supplementary data are available at *Bioinformatics* online.

## 1 Introduction

DNA methylation has been widely described to regulate gene expression in cancer (Chen *et al.*, 2019; Dor and Cedar, 2018; Ganesh *et al.*, 2020; Hu *et al.*, 2013; Maurano *et al.*, 2015). In general, hypermethylation is considered to decrease the activity of distinct regulatory elements, including proximal and distal regulatory regions, by reducing accessibility and transcription factor (TF) binding, and subsequent target gene expressions in various cancers (Aran *et al.*, 2013; Boyes and Bird, 1991; Chen *et al.*, 2020; Silva *et al.*, 2022; Yao *et al.*, 2015). DNA methylation at gene promoters and cytosine–phosphate–guanine (CpG) islands (CGIs) were originally recognized to be associated with gene expression levels (Klose and Bird, 2006). A large number of recent studies have focused on DNA methylation in more DNA element classes. For instance, it was reported that DNA methylation at enhancers, regulatory elements localized distal to the transcription start sites (TSS) (Thurman *et al.*, 2012), and the binding regions of ERα, FOXA1 and GATA3 is a breast cancer subtype-specific phenotypic feature (Fleischer, 2017). Cancer-specific differentially methylated CpG sites are enriched in known cancer genes and cell type-specific super-enhancers (SEs) (Yang *et al.*, 2017), comprising enhancer clusters in proximity (Hnisz *et al.*, 2013; Loven *et al.*, 2013). Moreover, methylation in the accessible chromatin defined by Assay for Transposase-Accessible Chromatin with high throughput sequencing (ATAC-seq) in primary human cancers was also reported to be involved in regulating distal target genes (Corces *et al.*, 2018; Lhoumaud *et al.*, 2019; Wang *et al.*, 2021). Meanwhile, the biophysical communication between genes and these regulatory elements could be explained using the 3D genome technique (Bonev and Cavalli, 2016; Dekker and Mirny, 2016; Rao *et al.*, 2014). In principle, the methylation status of cytosines on the ligated DNA is preserved in experiments on chromosome conformation capture (Li *et al.*, 2019). For an inferred CpG–target gene pair, mapping CpG and promoter simultaneously to 3D interaction anchors can help validate the pairs. Above all, annotating CpGs to these cell type-specific *cis*-regulatory elements and TF-binding sites (TFBS) helps explain target gene regulation based on DNA methylation in cancer.

At present, some databases for DNA methylation have been built. For example, MethHC is an integrated and web-based resource focusing on the aberrant methylomes of human cancer (Huang *et al.*, 2015, 2021). MethHC mainly provided aberrant DNA methylation in gene body, promoter and enhancer regions. MethMotif (Xuan Lin *et al.*, 2019) is a 2D TFBS database that records TFBS position weight matrices along with cell type-specific CpG methylation at TFBS. Other databases, such as MethyCancer (He *et al.*, 2008), MENT (Baek *et al.*, 2013), DiseaseMeth (Xiong *et al.*, 2017), DNMIV (Ding *et al.*, 2020), EWAS (Li *et al.*, 2019; Xiong *et al.*, 2020) and SurvivalMeth (Zhang *et al.*, 2021), have been developed for DNA methylation in diseases, especially cancer. These existing databases provide a resource for upstream *cis*-regulatory elements or TFBS information for CpGs or downstream annotations for target genes in different cells or diseases. They have become valuable resources for investigating DNA methylation. However, no existing databases comprehensively provide upstream and downstream annotations for CpG–gene pairs. In addition, the existing databases mainly focused on DNA methylation in gene promoter or body, ignoring distal *cis*-regulatory elements. Accumulating studies of DNA methylation within distinct distal *cis*-regulatory elements and the rapid accumulation of transcriptome and epigenomic profiles of human cancers have led to the urgent need to systematically collect the aforementioned *cis*-regulatory elements and TFs, besides integrating DNA methylation and gene expression profiles to explore DNA methylation regulation comprehensively.

We developed a comprehensive DNA methylation database CanMethdb to annotate CpGs and their target genes in diverse cancers. CanMethdb provides upstream *cis*-regulatory elements, especially those involving distal regions to genes, and TFBS annotations for CpGs. The *cis*-regulatory element includes cancer type-specific typical enhancers, SEs, chromatin accessibility regions defined by ATAC-seq, 3D chromatin interaction anchors and CGI. TFBS annotations include cancer type-specific ChIP-seq peaks and TF motifs. Furthermore, CanMethdb provides downstream functional and prognostic annotations for target genes of CpGs, computed using two methods by integrating abundant DNA methylation and gene expression profiles in 33 human cancers. In addition, 3D chromatin interaction verification analyses help users investigate whether the pairs are supported by 3D chromatin interaction anchors. Moreover, CanMethdb provides regulatory subnetwork analysis. The regulatory subnetwork reflects the relationship among CpGs, *cis*-regulatory elements, TFs and target genes. CanMethdb is an integrated cancer DNA methylation database that provides abundant gene regulatory annotations based on DNA methylation in multiple cancer types. CanMethdb may help biologists to perform in-depth studies of DNA methylation regulation mechanisms.

## 2 Data source and method

Our work was conducted with hg19 as the reference genome.

### 2.1 Data source

#### 2.1.1 Methylation and RNA-seq profiles

CanMethdb integrated DNA methylation (Illumina HumanMethylation450, 450K) profiles and FPKM upper quartile RNA-seq profiles for protein-coding genes of 33 The Cancer Genome Atlas (TCGA) (Tomczak *et al.*, 2015) cancer types to get CpG–target gene pairs. For RNA-seq profiles, gene ID conversion from the ensembl format to gene symbols for protein-coding genes was done according to GENCODE (Harrow *et al.*, 2012). For 450K probes, we screened CpGs in 22 autosomal chromosomes. For each DNA methylation profile, CpGs with a missing value (‘NA’) ratio of more than 10% were discarded. For the remaining CpGs, the missing values were imputed with the mean *β* values of samples in the same type (cancer or normal) (Dahlin *et al.*, 2016). For each gene expression profile, low-expression genes (FPKM = 0 for >10% of patients) were removed to ensure the reliability of the gene sets (Lee *et al.*, 2020). For each cancer type, only samples having both methylation and gene expression profiles were used to determine the target genes of CpGs.

#### 2.1.2 *Ci*s-regulatory elements

We collected and integrated five kinds of *cis*-regulatory elements to annotate DNA methylation: typical enhancers, SEs, ATAC-seq regions, 3D chromatin interaction anchors and CGI (Table 1). Typical enhancers in CanMethdb were downloaded from Fantom5 Transcribed Enhancer Atlas (Andersson *et al.*, 2014), a database describing enhancer regions defined using Cap Analysis of Gene Expression tags, encompassing 808 human samples, from which we manually annotated and screened 208 cancer cell line samples (Supplementary Table S1). SEs in CanMethdb were obtained from SEdb (Jiang *et al.*, 2019; Qian *et al.*, 2019) developed by our group using ROSE (Loven *et al.*, 2013) from 542 manually collected human public H3K27ac samples, from which we manually annotated and screened 315 cancer samples (Supplementary Table S2). The merged cancer type-specific ATAC regions were downloaded from a paper generating high-quality ATAC-seq data in 410 TCGA tumor samples across 23 cancer types (Corces *et al.*, 2018). We integrated the chromatin interaction datasets of 234 samples from 4Dgenome (Teng *et al.*, 2016), Oncobase (Li *et al.*, 2019), The 3D Genome Browser (Wang *et al.*, 2018) and NCBI (Barrett *et al.*, 2013), including 41 cancer samples (Supplementary Table S3). CGI was downloaded from UCSC (Haeussler *et al.*, 2019), involving 28 691 CGIs. CGI in CanMethdb had no distinction between diseases. SE samples covered 17 out of the 33 target gene cancer types (17/33), typical enhancers covered 25/33, ATAC-seq regions covered 23/33 and 3D chromatin interaction regions covered 12/33.

**Table 1.** Statistics of *cis*-regulatory elements and TFBS sources

*Cis*-regulatory elements and TFs	Sources	Number of all cancer samples^a^	Average number	Num. of TCGA cancer types
Typical enhancer	Fantom5	208	∼620 per sample	25
Super-enhancer	SEdb	315	∼500 per sample	17
ATAC-seq regions	Corces *et al.* (2018)	410	∼105 585 per cancer type	23
3D chromatin interaction	4DGenome Oncobase The 3D Genome Browser NCBI	41	∼74 392 per cell line	12
CGI	UCSC	–	28 691	–
TF by ChIP-seq	ENCODE Remap Cistrome ChIP-Atlas GTRD	1853	645 unique TFs	23
TF by motif	TRANSFAC MEME	–	More than 3000 motifs for ∼700 TFs	–

a Number of all cancer samples: the number of total cancer samples, including TCGA and non-TCGA cancer types.

#### 2.1.3 TF ChIP-seq peaks and motifs

We mapped CpGs to TFs based on ChIP-seq and motifs (Table 1). We manually screened 1853 cancer samples with TF ChIP-seq peaks (Table 1 and Supplementary Table S4) from ENCODE (The ENCODE Project Consortium, 2012), Remap (Cheneby *et al.*, 2018), Cistrome (Mei *et al.*, 2017), ChIP-Atlas (Oki *et al.*, 2018) and GTRD (Yevshin *et al.*, 2017). ChIP-seq peaks covered 23 out of the 33 target gene cancer types. We compiled 3000 DNA-binding motifs for ∼700 TFs, from TRANSFAC (Matys *et al.*, 2006) and Multiple Em for Motif Elicitation (MEME) suites (Bailey *et al.*, 2009; Saint-Andre *et al.*, 2016). TFs by motif annotations for the CpGs in CanMethdb had no distinction between diseases.

### 2.2 Method

#### 2.2.1 Inferring target genes for CpGs

We considered 20 nearby protein-coding genes (10 on each side) of the CpGs as the candidate set, which is recommended by the ELMER method (Yao *et al.*, 2015), one of the two methods we used to calculate the target genes for CpGs. ELMER have revealed regulatory roles for CpGs in enhancers, indicating the effect of DNA methylation on gene expressions can depend strongly on the genomic location of the CpG, and have been widely used to infer enhancer-target genes based on DNA methylation (Liu *et al.*, 2021; Qin *et al.*, 2022; Zhao *et al.*, 2021). If the gene number on one side was <10, then genes on the other side were used to replenish. The distance distribution statistics between CpGs to their 20 nearby genes showed that most of them (>90%) were located within 2 Mb of the CpGs (see details in the added ‘Statistics’ page on the website). To the best of our knowledge, this distance could cover promoter regions and most of the distal regulatory elements of genes (Chepelev *et al.*, 2012; Corces *et al.*, 2018; Hnisz *et al.*, 2013; Lieberman-Aiden *et al.*, 2009). Of the nearby 20 genes, whose expressions negatively correlated with the CpG methylation levels, were predicted as target genes. The genomic location annotation files of Illumina HumanMethylation450 BeadChip (450K, GPL13534) were downloaded from the GEO database (Barrett *et al.*, 2013), and probes on X and Y chromosome and single nucleotide polymorphism sites (ID start with ‘rs’) were removed; finally, 473 864 CpG probes remained. The gene TSS used here was extracted from the genome annotation file (hg19) in the GENCODE database (Harrow *et al.*, 2012).

Pearson’s correlation coefficient based on Python and R-based ELMER (Yao *et al.*, 2015) was used to determine target genes with CpG methylation and RNA-seq profiles. In each cancer type, we identified target genes from the candidate gene set for each CpG. For a candidate gene, if it was a target gene of a CpG with either one of the two methods, then it was stored as a target gene in CanMethdb. Pearson correlation coefficient in CanMethdb was implemented using Python with scipy.stats.pearsonr (Pearson’s correlation coefficient <0, Pearson’s *P*-value ≤0.05). ELMER is a bioinformatics tool that correlates the enhancer state with the expression of nearby genes based on methylation to identify transcriptional targets in cancer (Yao *et al.*, 2015). It uses a non-parametric *U*- test, namely the Wilcoxon test, to compare methylation quintiles versus the expression to determine the target genes of CpGs. For a CpG in one cancer type, ELMER ranked samples according to the CpG *β* values. Subsequently, the samples were assorted into two groups to screen target genes: the most unmethylated and the most methylated (we used the default 20%) to test whether the gene expression level of the former was greater than the level of the latter using the Wilcoxon test (*P*-value ≤0.05).

After computing, a total of 38 986 060 CpG–target gene pairs (ranging from 269 903 to 2 053 444 in different cancers) in 33 TCGA cancer types, were obtained. There were common pairs among different cancer types and 6 769 130 pairs were unique, involving 385 217 CpGs and 18 044 target genes.

Because recent studies showed that CpG methylation could also be positively related to target gene expression (Shin *et al.*, 2022), we also provided CpG–gene pairs with positive correlations (Pearson’s correlation coefficient >0, Pearson’s *P*-value ≤0.05). If users want to view positive pairs for different cancer types, they can obtain them from the download page of the database.

#### 2.2.2 Annotating CpGs to *cis*-regulatory elements and TFs

CpGs involved in CpG–target gene pairs were annotated to the aforementioned distinct *cis*-regulatory elements and TF ChIP-seq peaks in matched/all cancer types with bedtools (Quinlan and Hall, 2010). The genomic location version used here was hg19.

TF motif occurrences at CpG sites were measured against 21 bp sequences composed of cytosine and its nearby 20 bases (10 on each side), using Find Individual Motif Occurrence (Grant *et al.*, 2011) from the MEME suite (Bailey *et al.*, 2009). Finally, more than 160 000 CpGs were inferred to be bound by ∼300 TFs with a *q*-value threshold of 1e–3.

As a result, 229 699 out of the total 473 864 CpGs were mapped to SEs; 6167 to fantom5 enhancers; 84 410 to ATAC-seq regions; 388 626 to TFs by ChIP-seq; 374 660 to TFs by motif; 412 283 to chromatin interaction regions and 145 842 to CGIs.

#### 2.2.3 Sequence conservation

We obtained phyloP46way scores from multiple alignments of 46 mammal genomes from the UCSC browser to measure the conservation of each CpG.

#### 2.2.4 Differentially methylated CpGs

Limma is a widely used package, designed for differential expression analysis of data arising from microarray experiments (Smyth, 2005). It provides an integrated data analysis solution, using advanced computational algorithms to deliver reliable performance on large datasets and powers differential expression analyses for RNA-sequencing and microarray studies (Ritchie *et al.*, 2015). We used the Bioconductor package ‘limma’ (Release 3.7) to calculate differentially methylated CpGs between tumor and normal samples with the false discovery rate ≤0.05 and the difference of mean DNA methylation level between normal and tumor samples ≥0.1. If the methylation level of a CpG was higher in tumor samples than that in normal samples, we defined it as hypermethylated one, otherwise hypomethylated one (Yang *et al.*, 2017).

We used this method to define methylation status for the paired CpGs in 20 cancer types (the other 13 cancer types lacked normal samples). Finally, we found that an average of 35 105 CpGs in each cancer type (ranging from 4540 to 48 597) was hypermethylated and 43 638 (ranging from 10 186 to 88 680 in different cancers) were hypomethylated.

## 3 Database use and access

### 3.1 Overview of CanMethdb

**Fig. 1.**
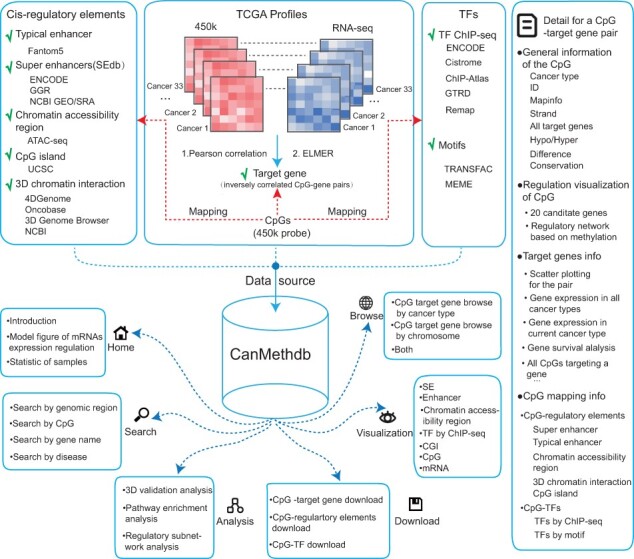
Database content and construction. CanMethdb contains abundant upstream and downstream annotations for genome-wide CpG–target gene pairs in diverse cancer types. CanMethdb provides a user-friendly interface to query, browse, analyze, visualize and download detailed information about these annotations

The main elements of CanMethdb, including data source and database use, are shown in Figure 1.

The current version of CanMethdb contains abundant annotations for genome-wide CpG–target gene pairs, including upstream *cis*-regulatory elements and TFBS annotations for the CpGs and downstream annotations for the target genes in 33 TCGA cancer types (Fig. 1). Especially, the correlations of the pairs are visualized using scatter plots, and the global correlation between the CpG and 20 nearby genes is also visible. In addition, 3D chromatin interaction verification analyses are provided to validate the pairs by mapping CpG and promoter simultaneously to 3D interaction anchors. Moreover, CanMethdb provides regulatory subnetwork analysis. Users can submit a genomic region list, TFs, CpGs or genes to extract the subnetwork (composed of input nodes and their one-step neighbors) from the CpGs to target genes, TFs and *cis*-regulatory elements background network. CanMethdb provides a user-friendly interface to query, analyze, browse, download and visualize detailed information about CpG–target gene pairs.

### 3.2 A Search interface for retrieving CpG–target gene pairs in different cancer types

**Fig. 2.**
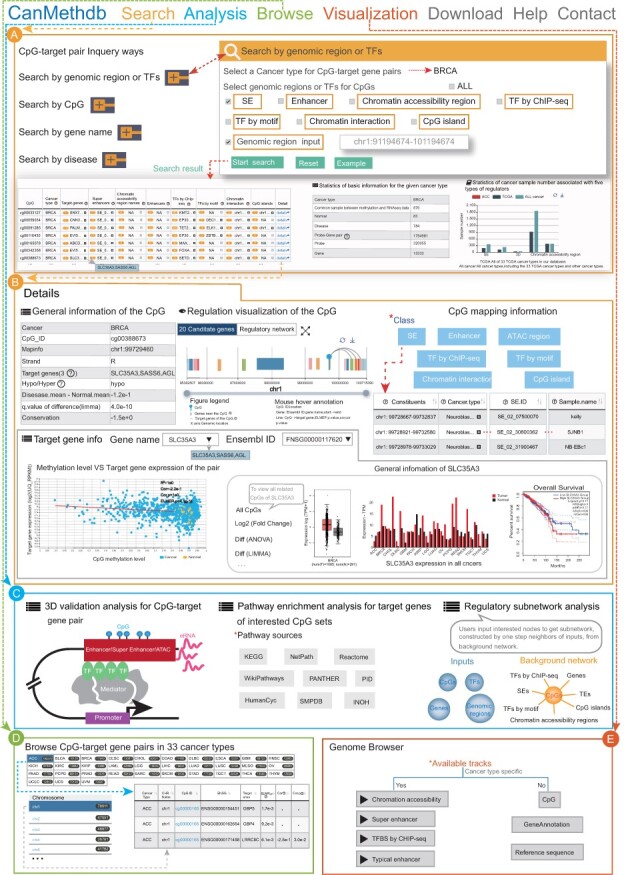
Main functions and usage of CanMethdb. (**A**) Four inquiry modes and search results including statistics of annotations for related CpGs, basic information of chosen cancer types and the number of cancer samples of *cis*-regulatory elements and TFs for cancer samples. (**B**) Details about upstream and downstream annotations for a CpG and its target genes. (**C**) Three online tools to interactively analyze the gene regulation, including ‘3D validation analysis’, ‘Pathway enrichment analysis’ and ‘Regulatory subnetwork analysis’. (**D**) Browsing details of CpG–target gene pairs in 33 cancers. (**E**) Genome browser to visualize the upstream *cis*-regulatory elements and TFs for CpGs and genes

CanMethdb provides four kinds of inquiry paths to get annotations of CpG–target gene (Fig. 2A). In ‘Search by genomic region’, in a cancer type, users can either input a genomic region or select one or more of the following seven categories of CpG annotation information: SE, enhancer, ATAC-seq regions, 3D chromatin interaction regions, CGI, TFs by ChIP-seq and TFs by the motif. In ‘Search by CpG’, users should input a CpG in 450K CpG name format. In ‘Search by gene name’, users can input a gene name to get paired CpGs. In ‘Search by disease’, users can select ‘hyper’ or ‘hypo’ to get all hyper- or hypomethylated CpGs of the selected cancer type. The information on the search results is displayed mainly in a table on the result page (Fig. 2A). Each row in the table describes a related CpG, target genes for the CpG in selected cancer type and all *cis*-regulatory elements and TFs annotated to the CpG in cancers. The search result page also provides basic information for the cancer type chosen and statistics of the cancer sample number of *cis*-regulatory elements and TF ChIP-seq.

Users can view detailed information for each CpG and its target genes on a new page by clicking ‘detail’ at the end of each row. The detailed information is listed below (Fig. 2B): (i) general information of the CpG. Basic information for the CpG, such as location, differences of methylation status between cancer and normal samples and conservation, is provided. (ii) Regulation visualization of the CpG. Users can intuitively visualize the global relationships between CpG and its surrounding 20 genes. The association degree between the CpG methylation level and target gene expression is shown by the line thickness. Besides, regulatory network provides an overview of the relationships between the CpG and its target genes, annotated *cis*-regulatory elements and TFs in cancers. (iii) Target genes information of the CpG. Users can view detailed information on the expression and survival of each target gene of the CpG. This includes a scatter plot to show the correlation between the CpG methylation levels and each target gene expression status, the expression patterns of target genes in 33 cancer types, and the expression pattern and survival analysis in selected cancer types. Besides, users can also click the gene name hyperlink to view all related CpGs of the gene in turn. (iv) CpG annotating information. Detailed mapping information for the CpG to *cis*-regulatory elements and TFs is provided in this section. Users can switch to the component of interest to view detailed information. Specifically, for typical enhancers, SE, ATAC-seq regions, 3D chromatin interaction anchors and TFs by ChIP-seq, users can choose only to view them in target gene matched cancer type or in all cancer samples by clicking the radio button.

### 3.3 Online analysis tools

CanMethdb provides the following three analyses to interactively analyze the gene regulation based on methylation (Fig. 2C): (i) 3D validation analysis. Users input CpGs or gene names directly to explore whether 3D chromatin interaction anchors support related CpG–target gene pairs in CanMethdb in different tissues. The input can also be genomic regions. Then, CpGs in these regions are explored. The related CpG–target gene pairs of inputs are analyzed using bedtools (Quinlan and Hall, 2010). The CpGs and gene promoters in pairs are analyzed separately against two anchors of all 3D chromatin interactions in the selected tissue. As a result, only the pairs with at least one end overlapping with an anchor are displayed. (ii) Pathway enrichment analysis. CanMethdb integrated 2880 pathways from 10 pathway databases: KEGG, Reactome, NetPath, WikiPathways, PANTHER, PID, Hu-manCyc, CTD, SMPDB and INOH (Cerami *et al.*, 2011; Kanehisa *et al.*, 2016). Users input CpG sets and select target gene cutoffs to perform pathway enrichment analysis with target genes. The output table contains CpG–target gene pairs. (iii) Regulatory subnetwork analysis. The components of the regulatory network include CpGs, cancer-specific target genes, SE, typical enhancers, ATAC regions, 3D chromatin interaction regions and TFs by CHIP-seq, as well as CGI and TFs by motif. The edges in the network link CpGs to other nodes. Users select a cancer type and input node names or regions. The node names can be any one or more of TFs, gene names or CpGs. If users input TFs, the mapped CpGs by ChIP-seq and motifs are shown on the results page. If users input gene names directly, the cutoff of target gene prediction could be set by users, and then paired CpGs are shown in the result. If users input CpGs, the target genes and mapped *cis*-regulatory elements and TFs would be shown. If users input regions, five kinds of regulatory elements (SEs, enhancers, chromatin accessibility regions and 3D chromatin interaction regions of the current cancer type as well as CGIs overlapping with the inputs) are shown. In addition, CpGs located in these elements and their target genes are also shown on the result page.

### 3.4 User-friendly browsing

This page is organized as an interactive table that allows users to quickly search for interested CpG–target gene pairs according to ‘chromosome’ and ‘cancer type’ and hence browse all CpG–target gene pairs more conveniently (Fig. 2D).

### 3.5 Data visualization

We developed a personalized genome browser with jBrowse to help users view transcriptional regulatory information of genes across the genome comprehensively and intuitively. TFs by ChIP-seq tracks and *cis*-regulatory element tracks, including typical enhancers, SE and ATAC-seq regions, were arranged by cancer type (Fig. 2E). CpG and gene tracks were also supported at the same time. Users could view regulatory information around a CpG, gene or genomic region of interest via data visualization.

### 3.6 Data download

All CpG–target gene pairs, including the negatively and positively correlated pairs, and CpGs mapping to five kinds of *cis*-regulatory elements and two kinds of TFs are provided on the ‘Download’ page. In addition, CanMethdb supported the export of query results on each search result page.

### 3.7 Case study

We searched the database with a reported CpG–target gene pair cg19942083–PTPN6 to illustrate the usage of CanMethdb by inputting the CpG name, CpG location or the gene name. The methylation of cg19942083, located in the intergenic region, was associated with kidney function and lower renal PTPN6 expression (Chu *et al.*, 2017). We inquired this pair in CanMethdb and found that PTPN6 was a target gene of cg19942083 in kidney cancer, which was consistent with the previous report. CanMethdb stored information on three types of kidney cancer, including kidney chromophobe, kidney renal clear cell carcinoma (KIRC) and kidney renal papillary cell carcinoma. Taking KIRC as a case, we retrieved more annotations for this pair in CanMethdb. We found that the hypomethylation of cg19942083, located within the SE region, might recruit TFs, such as FOXA1, MYC and EGR1, to the CpG site and lead to the overexpression of PTPN6 through interacting with its promoter.

**Fig. 3.**
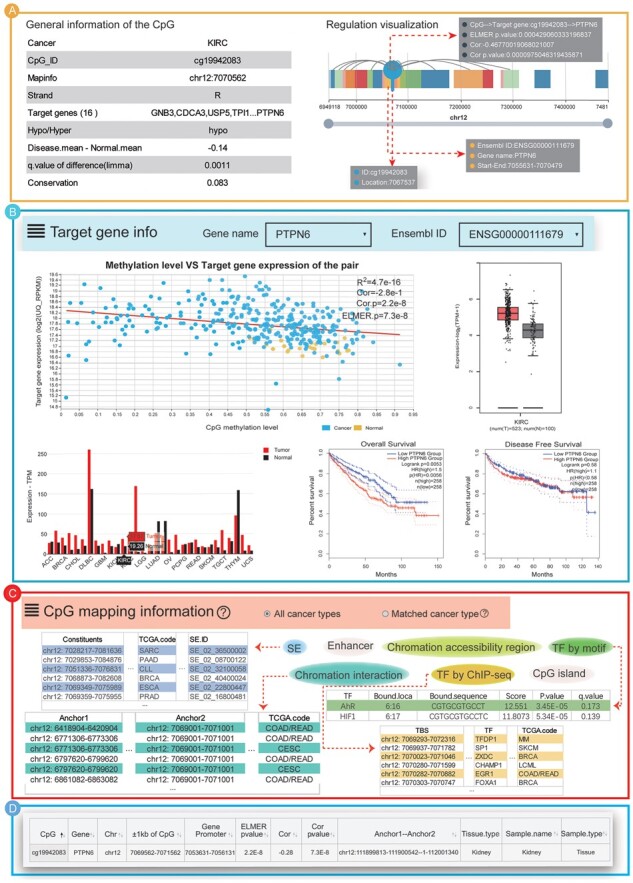
Case study with cg19942083–PTPN6 pair reported in kidney. (**A**) PTPN6 was significantly targeted by cg19942083 in KIRC and cg19942083 was located distal to PTPN6. (**B**) PTPN6 was associated with the prognosis of KIRC and was overexpressed in most cancers including KIRC. (**C**) The cg19942083 was located within SE regions and bound by multiple TFs, such as FOXA1 and EGR1, in cancers. (**D**) The 3D chromatin interaction validation analysis showed that the pair cg19942083–PTPN6 was located in a 3D chromatin interaction loop in kidney tissues

In detail, first, PTPN6 was significantly targeted by cg19942083 (Pearson’s correlation coefficient =–2.8, Pearson’s *P*-value =2.28E-8; ELMER’s *P*-value = 7.3E-8) in KIRC (Fig. 3B), where cg19942083 was significantly hypomethylated (Fig. 3A) and PTPN6 was overexpressed (Fig. 3B). The general and regulation visualization information for CpG showed that cg19942083 was located distal to PTPN6 (∼4.9 kb downstream from the TSS of PTPN6) (Fig. 3A). Moreover, the upstream annotation for the CpG showed that cg19942083 was located within SE regions and bound by multiple TFs, such as FOXA1 and EGR1, in cancer samples (Fig. 3C). In addition, the downstream annotation for the target gene showed that PTPN6 was associated with the prognosis of KIRC and was also overexpressed in most other cancer types (Fig. 3B). Interestingly, the 3D chromatin interaction validation analysis showed that the pair cg19942083-PTPN6 was located in a 3D chromatin interaction loop in kidney tissues (Fig. 3D). Furthermore, studies showed that PTPN6 was a cancer marker gene closely related to DNA methylation. These results prompted that the DNA methylation of distal *cis*-regulatory elements might play an important role in the dysregulation of target PTPN6 expression through TF binding and biophysical communication in KIRC. Thus, we concluded that CanMethdb was a valuable platform for performing in-depth research and revealing DNA methylation regulation mechanisms in cancer.

### 3.8 Availability

The core codes, we have used to generate CpG–gene pairs and differentially methylated CpGs are freely available in the GitHub repository (https://github.com/chunquanlipathway/CanMethdb). To allow researchers programmatically query and analyze CpG–target gene pairs in CanMethdb, we provided web APIs interfaces. CanMethdb APIs are protocols that allow researchers to query a CanMethdb data resource in the RESTful API format. We have added an ‘API’ page in the website, where we provided seven API interfaces, including four query and three analysis interfaces, as well as detailed parameter descriptions.

## 4 Discussion

The emerging importance of DNA methylation in upstream *cis*-regulatory elements and TFs increases the need for comprehensively understanding DNA methylation regulation in human cancers. In general, methylation is considered to regulate the activation of distinct regulatory elements by reducing accessibility and TF binding. Some databases, such as MethyCancer (23), MENT (24), DNMIV (26), EWAS (27, 28), MethHC (Huang *et al.*, 2015, 2021) and SurvivalMeth (29), have been developed for DNA methylation. These databases provide a resource for upstream or downstream annotations for DNA methylations and target genes in different cells or diseases and have become valuable resources for investigating DNA methylation. However, no existing databases comprehensively provide upstream and downstream annotations for CpG–gene pairs in cancers and they mainly focused on DNA methylations in gene promoter or body, ignoring distal *cis*-regulatory element (Table 2). In recent years, an increasing number of studies have focused on DNA methylation located in distal regulatory elements, away from gene promoter and body in cancers. The increasing studies of DNA methylation within distinct distal *cis*-regulatory elements as well as the rapid accumulation of transcriptome and epigenomic profiles of human cancers have led to the urgent need to systematically collect distinct *cis*-regulatory elements involving distal regions and TFs, besides integrating methylome and transcriptome profiles to explore DNA methylation regulation comprehensively. We developed CanMethdb, which focused on providing comprehensively distinct upstream regulatory elements, especially those involving regions distal to genes and TF annotations for DNA methylation and downstream target genes of DNA methylation as well as related annotations in diverse cancers.

**Table 2.** Comparison of information in CanMethdb with other databases

Item	CanMethdb	SurvivalMeth	DNMIVD	EWAS	MethHC2.0	MENT	MethyCancer
Distal elements	4^a^	1	1	–	1	–	–
TFs to CpGs by ChIP-seq	√	–	–	–	–	–	–
TFs to CpGs by motif	√	–	–	–	–	–	–
CpG–gene pair sources	2^b^	–	1	Unknown	–	1	–
3D validations	√	–	–	–	–	–	–
Subnetwork analysis	√	–	–	–	–	–	–
Pathway sources	10^c^	–	1	–	1	–	Unknown
Number of cancers	33	36	26	39	33	30	10
Number of target genes for CpGs	18 044	∼20 000	20 141	36 397	28 047	15 284	7100

a *Cis*-regulatory elements involving regions distal to genes include super-enhancer, enhancer, ATAC-seq regions and 3D chromatin interaction archors.

b CpG–gene pair sources were computed from Pearson correlation and Wilcoxon test.

c Pathway were collected from 10 resources: KEGG, Reactome, PANTHER, SMPDB, NetPath, PID, HumanCyc, CTD, WikiPathways and INOH.

CanMethdb supported a user-friendly interface to query, analyze, browse, visualize and download detailed information on DNA methylation regulation. The main advantages of CanMethdb were as follows: (i) it provided distinct upstream annotations for CpGs, including cancer type-specific *cis*-regulatory elements, TFs and CGI. (ii) It provided CpG–target gene pairs, computed by two methods through integrating abundant DNA methylation and gene expression profiles in 33 human cancers. Especially, the correlations of the pairs were visualized using a scatter plot; the global correlations between the CpG and the 20 nearby genes were also visible. In addition, 3D chromatin interaction verification analyses were provided to validate the pairs by mapping CpG and promoter simultaneously to 3D interaction anchors. (iii) It provided expression patterns survival and pathway annotations for downstream target genes of CpGs. (iv) It provided regulatory subnetwork analysis. Users could submit a genomic region list, TFs, CpGs, or genes to extract subnetwork (composed of input nodes and their one-step neighbors) from the CpGs to target genes, TFs by ChIP-seq, TFs by motif, SE, enhancers, ATAC-seq regions, 3D chromatin interaction regions and CGI background network. The regulatory subnetwork reflected the relationship among *cis*-regulatory elements, TFs, CpGs and genes. (v) It provided four user-friendly ways to retrieve CpG–target gene pairs, including ‘by genomic region’, ‘by CpG’, ‘by gene name’ and ‘by disease’. (vi) Users could browse and download all CpG–target gene pairs as well as CpGs to distinct *cis*-elements and TF pairs in CanMethdb. (vii) Users could view regulatory information around a CpG, gene or a functional region of interest in a specified cancer type via data visualization.

The fields of epigenome and transcriptome analyses have developed rapidly and become highly investigated areas. CanMethdb is a novel DNA methylation database comprising a systematic integration of abundant *cis*-regulatory elements, especially those involving distal regions to genes, and TFBS, in addition to a large number of DNA methylation and gene expression profiles to comprehensively annotate genome-wide CpG–target gene pairs in cancers. Our efforts to establish this database were prompted by the need for researchers to understand the functions of methylations, especially methylation sites falling within distal regulatory elements and TFBS in cancers. These researchers include cell/molecular biologists, geneticists and data scientists. CanMethdb can help to understand the mechanism of gene regulation and the pathology of cancers, even in biological and clinical studies. For example, DNA methylation is promising in the application of liquid biopsy-based cancer diagnosis (Dor and Cedar, 2018; Yu *et al.*, 2020). CanMethdb provides abundant annotations for genome-wide DNA methylation in cancers. Thus, researchers can screen key CpGs and related genes, important chromatin regions, or TFs in different cancer types with CanMethdb to facilitate in-depth research in identifying putative biomarkers in cancer diagnoses. Continuous efforts will be made to update the platform with available data and improve the functional properties of the CanMethdb database.

## Supplementary Material

btac783_Supplementary_Data
